# Calcium-dependent protein kinase OsCPK10 mediates both drought tolerance and blast disease resistance in rice plants

**DOI:** 10.1093/jxb/erx145

**Published:** 2017-05-02

**Authors:** Mireia Bundó, María Coca

**Affiliations:** Centre for Research in Agricultural Genomics (CRAG), CSIC-IRTA-UAB-UB, Edifici CRAG, Campus de la UAB, Bellaterra, Barcelona, Spain

**Keywords:** Blast disease, calcium-dependent protein kinases, catalase, drought, oxidative damage, rice, ROS

## Abstract

Plant growth and productivity is negatively affected by different stresses. Most stresses trigger calcium signals that initiate acclimation responses in plants. The multigene family of plant calcium-dependent protein kinases (CPKs) functions in multiple stress responses by transducing calcium signals into phosphorylation events. This work reports that the OsCPK10 isoform positively mediates tolerance to different stresses in rice plants by enhancing their antioxidant capacity and protecting them from reactive oxygen species (ROS) damage, with the uncontrolled generation of ROS being a common feature of these stresses. Here, we show that the constitutive accumulation of an HA-tagged OsCPK10 full-length protein enhances the hydrogen peroxide detoxifying capacity of rice plants during desiccation. This is achived by modulating the accumulation of catalase proteins, which reduces the extent of lipid peroxidation and protects the integrity of cell membranes, resulting in drought tolerance. OsCPK10HA accumulation also confers blast disease resistance by interfering with fungal necrotrophic growth via a reduction in the accumulation of hydrogen peroxide. Furthermore, we show by bimolecular complementation assays that OsCPK10 is a plasma membrane protein that physically interacts *in vivo* with catalase A. OsCPK10 therefore appears to be a good molecular target to improve tolerance to abiotic stresses as well as to blast disease, which limit rice crop productivity.

## Teaser

OsCPK10 mediates drought tolerance and blast disease resistance in rice plants by enhancing their reactive oxygen species scavenging capacity and so reducing oxidative damage associated with both stresses.

## Introduction

Rice is the staple food for half of the world population. Unfortunately rice, as with other crops, is exposed to numerous environmental stresses that constrain its growth and development, culminating in harvest losses. Blast disease, caused by the ascomycete fungus *Magnaporthe oryzae*, is one of the most devastating diseases affecting rice crop worldwide (Dean *et al.*, 2012). This fungus can infect all plant parts provoking its death in juvenile stages or causing total grain loss in panicle infections. Annual losses of rice grain due to blast disease vary between 10–35% (Skamnioti and Gurr, 2009). Another important adverse factor for rice cultivation is drought. Around 75% of rice is cultivated in irrigated ecosystems (Bouman *et al.*, 2007). Water deficits affect rice growth and development during its entire life cycle but is more damaging during the reproductive and grain filling phases (Farooq *et al.*, 2012). Drought stress is a growing problem worldwide, affecting 50% of global rice production every year (Mackill *et al.*, 2010). Nowadays, in the context of global climate change, these negative environmental factors may be more pronounced and damaging. For this reason, combined with the global population increase, the study of rice plant responses against stresses is critical for developing new strategies to mitigate the effects of the upcoming environmental problems and to guarantee food security.

In the course of evolution, plants have evolved sophisticated strategies to adapt and survive stressful conditions. These strategies rely on an intricate signaling network in which calcium signals and phosphorylation cascades are important messengers. Calcium-dependent protein kinases (CDPKs or CPKs) are plant proteins involved in responses to both abiotic and biotic stimuli but also in growth and development (Asano *et al.*, 2012*a*; Boudsocq and Sheen, 2013; Schulz *et al.*, 2013; Romeis and Herde, 2014). These proteins combine within a single polypeptide chain, a calmodulin domain with four EF-hand Ca^2+^ binding motifs and a kinase domain, which gives them the ability to perceive Ca^2+^ fluctuations and rapidly translate them into a phosphorylation signal (Harmon *et al.*, 2001; Harper *et al.*, 2004). They are members of multigenic families for which functional diversification has been proposed (Harmon *et al.*, 2000; Ludwig *et al.*, 2004). Supporting this idea, CPK isoforms showed different subcellular locations, including the plasma membrane, cytosol, endoplasmic reticulum, peroxisomes, oil bodies, the nucleus and chloroplasts (Lu and Hrabak, 2002; Dammann *et al.*, 2003; Coca and San Segundo, 2010; Campos-Soriano *et al.*, 2011; Asai *et al.*, 2013; Campo *et al.*, 2014), as well as different expression patterns in plant tissues and in response to different stimuli (Harmon *et al.*, 2000; Ludwig *et al.*, 2004; Asano *et al.*, 2005; Ray *et al.*, 2007; Boudsocq and Sheen, 2013).

Several CPKs have been shown to be involved in stress tolerance in various plants and are especially well characterized in Arabidopsis (Mori *et al.*, 2006; Zhu *et al.*, 2007; Boudsocq *et al.*, 2010; Coca and San Segundo, 2010; Dubiella *et al.*, 2013; Zou *et al.*, 2015). The rice genome contains 31 different *OsCPK* genes (Asano *et al.*, 2005; Ray *et al.*, 2007). Functional studies using gain-of and loss-of-function strategies in rice plants have reported the participation of *OsCPK* genes in tolerance to abiotic stresses, namely the *OsCPK4*, *OsCPK9*, *OsCPK12*, *OsCPK13* and *OsCPK21* (Saijo *et al.*, 2000; Asano *et al.*, 2011, 2012*b*; Campo *et al.*, 2014; Wei *et al.*, 2014), as well as to biotic stresses, namely *OsCPK4* and *OsCPK10* (Fu *et al.*, 2013; Bundó and Coca, 2016). However, the precise mechanisms underlying OsCPK-mediated stress tolerance, as well as the components acting downstream of OsCPKs remains largely unknown. A better understanding of OsCPK functions is relevant to the genetic improvement of agronomically valuable traits, since these proteins are components of complex regulatory networks affecting other stress acclimation responses and developmental processes. This study reports that OsCPK10 functions to enhance the hydrogen peroxide (H_2_O_2_) detoxifying capacity of rice plants through modulating catalase protein accumulation, reducing lipid peroxidation levels, and preserving cell membrane integrity. Together this confers rice plants with improved drought tolerance under OsCPK10 constitutive accumulation. Moreover, OsCPK10 mediates blast disease resistance by reducing H_2_O_2_ accumulation during fungal necrotrophic growth. Furthermore, we show the *in vivo* interaction of OsCPK10 with catalase A, which could explain the elevated H_2_O_2_ detoxifying capacity against oxidative damage.

## Materials and methods

### Plant materials, growth conditions, and stress treatments

Rice (*Oryza sativa* var. Nipponbare) was grown at 28 °C in a 14h light/10h dark photoperiod. For drought stress treatments, plants were grown in sealed jars at 100% humidity for 10 days and left to air-dry for the required period of time. Abscisic acid (ABA) treatments were also conducted with 10 day-old seedlings by adding a 100 µM solution of ABA. Three technical and biological replicates were analyzed in each treatment.

### Production of transgenic rice plants

For the expression of the *OsCPK10HA* gene, we obtained the full-length *OsCPK10* coding sequence extended at the C-terminal with the sequences encoding the HA epitope. This DNA fragment was generated by PCR amplification from the Rice Genome Resource Center clone J013164K19, using the primers indicated in Supplementary Table S1 at *JXB* online, which introduced a *BamH*I restriction site at the 5′end (forward primer) and a *Sma*I restriction site and the HA-epitope sequences at the 3′end (reverse primer) just before the stop codon of the cDNA. The PCR fragment was cloned into the *BamH*I and *Sma*I sites of a pCAMBIA1300-derived vector containing the maize *Ubiquitin1* promoter (pUbi) and the *Nopaline Synthase* terminator (Nos-t) previously described (Campo *et al.*, 2014). The derived construct was verified by DNA sequencing. *Agrobacterium tumefaciens* strain EHA105 was transformed with the final vector for rice transformation and transgenic rice plants were produced as previously described (Sallaud *et al.*, 2003). Hygromycin resistance encoded in the T-DNA region was used to select the hemizygotic plants in *in vitro* culture. Those plants growing in the selective media were then transferred to soil pots for subsequent assays or for harvesting seeds. The transgene insertion copies were estimated by quantitative PCR using the *Sucrose Phosphate Synthase* reference gene as previously described (Yang *et al.*, 2005; Bundó *et al.*, 2014).

### RNA isolation and qRT-PCR analysis

Total RNA was extracted using TRIzol reagent (Life Technologies, www.thermofisher.com). DNAse treated RNA (1 µg) was retrotranscribed using the Transcriptor First cDNA Synthesis kit (Roche, www.lifescience.roche.com). qRT-PCR analyses were carried out in 96-well optical plates in a LightCycler® 480 System (Roche) according to the following program: 10 min at 95 °C, 45 cycles of 95 °C for 10 s and 60 °C for 30 s, and an additional cycle of dissociation curves to ensure a unique amplification. The reaction mixture contained 5 µl of SYBR Green Master mix reagent (Roche), 2 µl of 1:4 diluted cDNA samples and 300 nM of each gene-specific primer (Supplementary Table S1) in a final volume of 10 µl. The results for the gene expression were normalized to *OsUbi5* (LOC_Os01g22490). Three technical replicates were done for each sample.

### Protein extracts and immunoblot analysis

Protein extracts for OsCPK10HA immunodetection were obtained from a pool of at least four different plants as previously described (Bundó and Coca, 2016). For catalase immunodetection, the protein extracts were obtained from the soluble fractions after centrifugation of shoot samples, resuspended in two volumes of extraction buffer (50mM sodium-phosphate buffer at pH7, 1mM EDTA, 1% w/v insoluble polyvinyl-polypyrrolidone). Western blot analyses were performed using anti-HA (Sigma, www.sigmaaldrich.com) and anti-catalase (Abcam Ab1877, www.abcam.com) antibodies.

### Drought tolerance assays

The drought tolerance of soil-grown rice plants was evaluated as the recovery rate after an extreme drought treatment, where water was withheld until soil moisture was at the detection limit of a HH2 Moisture meter 2.3 (Delta-T devices Ltd.). Three independent assays were performed, with five plants per line. Water loss was evaluated by air-drying 10-day old seedlings and calculated as percentage using the following formula: (lost weight/initial weight) × 100. Three biological and technical replicates in three independent assays were performed. Malondialdehyde (MDA) content and relative electrolyte leakage was determined as described in (Campo *et al.*, 2014), using pools of three biological replicates of control and air-dried 10-day old seedlings.

### Determination and detection of hydrogen peroxide content

The H_2_O_2_ content in control or air-dried shoots was determined as described in Velikova *et al.*, 2000. Briefly, 500 mg of frozen and pulverized shoots were homogenized in 300 μl of 0.1% (w/v) trichloroacetic acid and centrifugated for 15 min at 13,000xg. A 500 μl volume of the recovered supernatant was mixed with 500 μl of 10 mM sodium-phosphate buffer at pH 7.5 and 1 ml of 1M potassium iodide. The absorbance at 390 nm was then measured. The H_2_O_2_ concentration was calculated using de-extinction coefficient ε=0.28 µM^−1^ cm^−1^. Accumulation of H_2_O_2_ was detected by *in situ* oxidation of diaminobenzidine as previously described (Thordal-Christensen *et al.*, 1997).

### Oxidative stress tolerance assays

Tolerance assays to the oxidative agent methylviologen (MV, Sigma) were done with leaf fragments of 10-day old seedlings. Leaf fragments were incubated for 4 d at 28 °C, with a 16 h light/8 h photoperiod at a light intensity of 200 lux in sterile water (mock) or 10 μM MV solution (MV treatment). Both solutions were supplemented with 0.02% Tween-20. At the end of the assay, chlorophyll content was measured as described (Lichtenthaler and Buschmann, 2001).

### Rice blast disease resistance assays

*M. oryzae* infections with the FR13 strain (provided by Dr. D. Tharreau, CIRAD, Montpellier France) were performed using the whole plant infection assay. *M. oryzae* infections with Guy11-GFP (provided by Dr. A. Sesma, GBGP Madrid, Spain) were performed using the detached leaf infection assay, as previously described (Bundó and Coca, 2016).

### Subcellular localization and bimolecular fluorescence complementation analysis

To create an *OsCPK10-GFP* fusion gene, the *OsCPK10* coding sequence without the stop codon was amplified by PCR using primers listed in Supplementary Table S1, which introduced *Eco*RI and *Not*I restriction sites at the 5’ and 3’ ends, respectively. This DNA fragment was cloned into the pENTR3C plasmid (Invitrogen). *OsCPK10* was then recombined into the Gateway binary destination vector pMDC85 (Curtis and Grossniklaus, 2003). Similarly, the *OsCATA* coding sequence without the stop codon was amplified by PCR, cloned into the pENTR3C vector as an *Eco*RI-*Eco*RV fragment and then recombined into the pEarlyGate102 vector (Earley *et al.*, 2006) to create an *OsCATA-CFP* fusion gene. For the BiFC constructs, *OsCPK10* and *OsCATA* coding sequences were recombined from the pENTR3C derived plasmids into the pXNGW and pXCGW vectors (courtesy of Wolf Frommer, (Kim *et al.*, 2009).

The fusion genes were transiently expressed in *Nicotiana benthamiana rdr6IR* mutant leaves (Schwach *et al.*, 2005) by agroinfiltration using the *A. tumefaciens* strain EHA105 as previously described (Campo *et al.*, 2013). Observations were performed 48 hours after infiltration. Confocal laser scanning analysis was performed using a Leica TCS-SP5II microscope. Reconstituition of YFP and GFP were detected through excitation with an Argon ion laser emitting at 488 nm and detection of fluorescence at 500–600 nm. Chlorophyll autofluorescence was detected at 650–750 nm. CFP was excited with a 458 nm laser and detected at 465–600 nm. To confirm plasma membrane localization, leaf cells were plasmolysed with 0.75 M mannitol for 15 min or stained with a 10 µM solution of the lipophilic dye FM4-64 (Molecular Probes). Fluorescence was observed immediately after washing by exciting with a 543 nm argon ion laser.

## Results

### OsCPK10 expression is induced by both biotic and abiotic stresses in rice plants

**Fig. 1.**
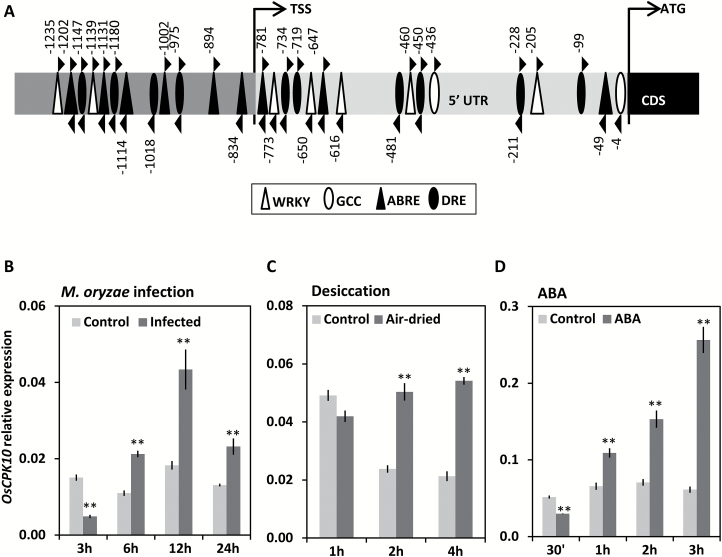
*OsCPK10* expression in response to biotic and abiotic stress in rice plants. (A) Diagram of the *OsCPK10* promoter region showing the position of the biotic and abiotic stress-related *cis*-elements. (B–D) *OsCPK10* expression in response to *M. oryzae* infection of 10^5^ spores/ml at the indicated period of time post infection (B), in reponse to air-drying stress (C), or in reponse to 100 µM ABA treatment (D). *OsCPK10* transcript levels were determined by qRT-PCR and normalized to *OsUbi5* mRNA levels. Values represent means and standard deviations of three replicates. Asterisks denote significant differences, one-way ANOVA and Tukey′s test, ** *P*≤0.01.

A search for altered gene expression in rice leaves in response to *M. oryzae*, using a previously described microarray global transcriptomic analysis (Campo *et al.*, 2013), identified *OsCPK10* as upregulated at 30 min after treatment, with a fold change of 1.32, *P*=0.031. This data suggests that *OsCPK10* might be involved in the defense response of rice plants. A detailed analysis of *OsCPK10* promoter sequences, lying 1375 bp upstream of the coding sequence at the end of previous locus LOC_Os3g57430, identified several stress-responsive regulatory elements that are known to contribute to the expression of stress-related genes at a transcriptional level (Fig. 1A, Supplementary Table S2). Among them, there are response elements to biotic stress, namely GCC and W boxes, but also to abiotic stress. The most frequently found elements were ABRE, which are ABA-responsive, and DRE, which are dehydratation-responsive. ABRE and DRE are important interdependent regulatory elements of gene expression in response to dehydratation stress (Narusaka *et al.*, 2003). These observations suggest that *OsCPK10* expression might also be regulated by abiotic stress.

Consistently, gene expression analyses showed that *OsCPK10* was regulated in rice leaves in response to *M. oryzae* fungal infection (Fig. 1B). *OsCPK10* transcript levels reached maximum levels at 12 hours post infection (hpi), with a 2.4 fold induction coinciding with the invasive growth and ramification of *M. oryzae* into the foliar epidermal cells (Kankanala *et al.*, 2007; Wilson and Talbot, 2009; Campos-Soriano *et al.*, 2013). Thus, *OsCPK10* expression correlates with pathogen growth into the rice plants.

*OsCPK10* transcript accumulation was also monitored in rice plants in response to desiccation stress. A 2.5 fold induction of *OsCPK10* transcripts was maintained over time in rice leaves under air-drying stress, which differed from the transient induction detected at 1 h in the control leaves that was probably due to drought stress induced by opening the plant containers (Fig. 1C). Given that several ABRE motifs were identified in the *OsCPK10* promoter, *OsCPK10* expression was also monitored in response to treatment with ABA. This phytohormone is a well-known and important signal during drought stress responses in plants, with many drought stress-inducible genes containing the related *cis*-regulatory elements in their promoters (Qin *et al.*, 2011). Consistent with the presence of ABREs, a marked accumulation of *OsCPK10* transcripts occurred in rice plants in response to ABA (Fig. 1D). *OsCPK10* induction reached a maximum of 4.2 fold at 3 h of treatment. Together, these results show that *OsCPK10* is induced by both biotic and abiotic stress, suggesting that this gene might be involved in responses to different stresses in rice plants.

### OsCPK10HA protein accumulates in transgenic rice plants

**Fig. 2.**
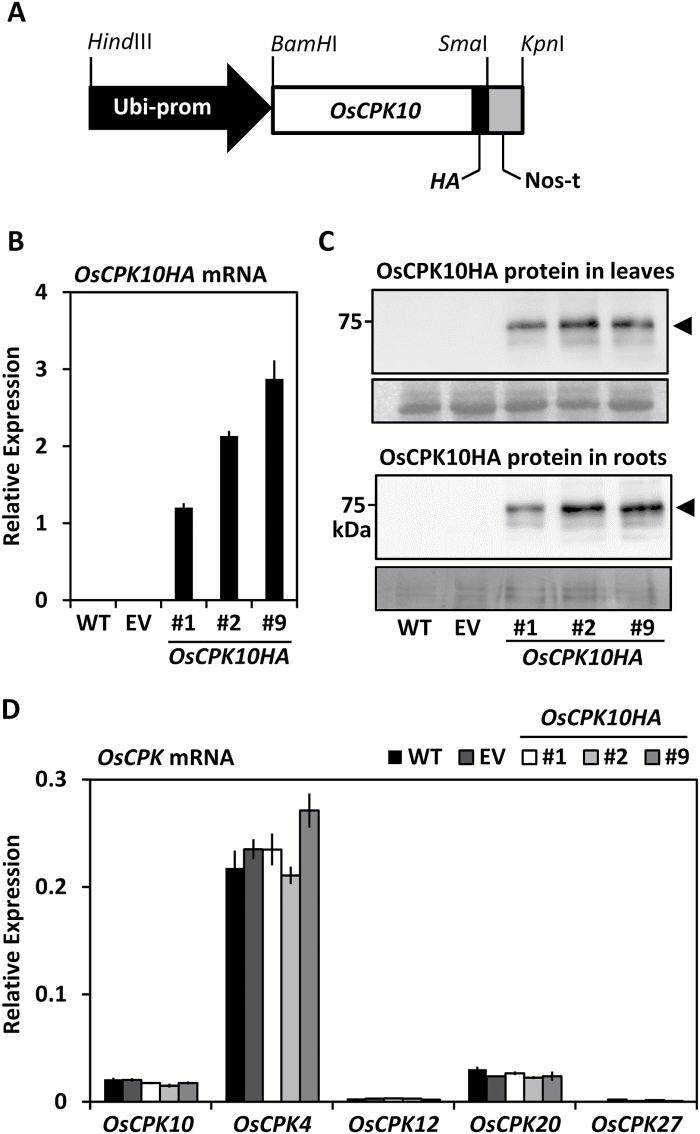
OsCPK10HA accumulation in transgenic rice plants. (A) Schematic representation of the p*Ubi*:*OsCPK10HA:nos* transgene used for rice transformation. (B) Transcript levels of *OsCPK10HA* in leaves of wild-type (WT), empty vector (EV) and the indicated *OsCPK10HA* lines as determined by qRT-PCR analysis using *OsUbi5* mRNAs for normalization. Values are means and standard deviations of three replicates. (C) OsCPK10HA protein accumulation in leaves and roots as determined by western blot analysis using specific anti-HA antibodies. Lower panels show the loading control for leaves, 25 μg per lane, using Ponceau staining, and for roots, 35 μg per lane, using Coomassie Blue staining. (D) Transcript levels of endogenous *OsCPK10* and different *OsCPK* family members in leaves as determined by qRT-PCR analysis using *OsUbi5* mRNAs for normalization. Values are means and standard deviations of three replicates.

To characterize the biological function of *OsCPK10*, transgenic rice plants overexpressing *OsCPK10* were generated. A pCAMBIA 1300-derived vector containing the *OsCPK10* full-length cDNA extended at C-terminal with the HA epitope sequences, under the control of the maize *Ubiquitin1* promoter and the *Nopaline Synthase* terminator, was used for transformation (Fig. 2A). The construct was designed for the production of a full-length OsCPK10 protein preserving its regulatory domains, namely the junction and calmodulin domains, and was HA tagged at C-terminal to avoid potential interference with the N-terminal localization signals. Five independent transgenic lines were obtained that accumulated the recombinant protein as determined by immunoblot analysis (Supplementary Fig. S1), and three of them, lines #1, #2 and #9, were selected to obtain homozygous lines in the progeny plants. However, no homozygous lines could be identified, either in T_2_ or in T_3_ generation plants. All the selected lines contained a single transgene insertion as estimated by qPCR in comparison with the *Sucrose Phosphate Synthase* gene and segregation ratios in hygromycin selection media were about 50% of resistant plants.

Quantitative RT-PCR analysis confirmed that *OsCPK10HA* transcripts accumulated in the hemyzygous plants in the T_3_ generation (Fig. 2B). Moreover, the tagged protein was detected by immunoblot analysis in the roots and shoots of the selected plants (Fig. 2C). These results show that the generated transgenic lines expressing *OsCPK10HA* accumulate the encoded protein in their tissues. Additional qRT-PCR analysis showed that the constitutive expression of *OsCPK10HA* did not modify *OsCPK10* endogenous expression or other related *OsCPK* genes (Fig. 2D).

*OsCPK10HA* plants showed a normal phenotypic appearance quite similar to control plants when grown under greenhouse conditions (Supplementary Fig. S2A). Several growth parameters were measured in three independent assays with plants randomly distributed and no statistically significant differences were observed among *OsCPK10* and control plants. They flowered at the same time (Supplementary Fig. S2B), reached the same height at heading time (Supplementary Fig. S2C), produced similar grain yield (Supplementary Fig. S2D) and had similar seed weight (Supplementary Fig. S2E). The expression of *OsCPK10HA* therefore appears not to have deleterious effects on rice plant performance.

### OsCPK10HA expression improves drought tolerance in rice plants

**Fig. 3.**
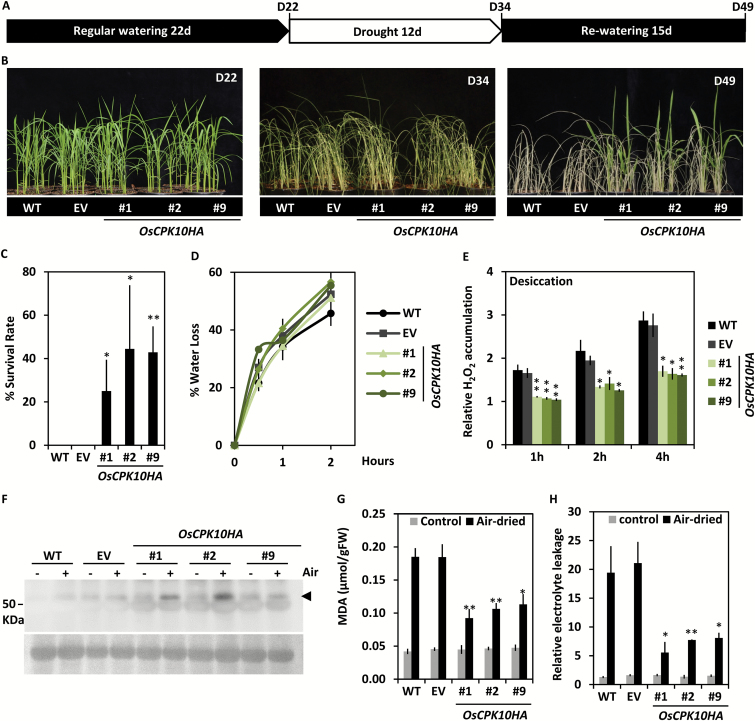
Drought tolerance of *OsCPK10HA* rice plants. (A) Diagram of the experimental design for drought tolerance assays. (B) Phenotypical appearance of wild-type (WT), empty vector (EV) and three *OsCPK10HA* lines (#1, #2, #9) at the indicated phases of the drought tolerance assay. (C) Plant survival rate after rewatering (D49). Values are the means ± standard error of the mean, of three independent assays with five plants per line. (D) Water loss rate of air-dried 10 day-old seedlings, with 9 seedlings per line. (E) H_2_O_2_ content in air-dried seedling shoots at the indicated times (h). (F) Western blot analysis of catalase (arrow) accumulation in control (-) or air-dried rice shoots (+) for 2 h. Lower panel corresponds to Ponceau staining of the membrane as a loading control. (G) MDA content, and (H) relative electrolyte leakage of seedling shoots under control conditions or after 8 h or 4 h of air-drying treatment, respectively. Values are the means ± standard error of the mean, of three biological replicates from a pool of three seedlings and three independent experiments. Asterisks show significant differences, one-way ANOVA analysis and Tukey test, *P≤0.05, **P≤0.01. (This figure is available in colour at *JXB* online)

To investigate the role of OsCPK10 in the acclimation of rice plants to water deficit stress, the *OsCPK10HA* plants were assessed for drought tolerance. Transgenic, empty vector (EV) and wild-type plants were grown under a fully watered regime for 22 d (Fig. 3A, B, D22) and then deprived of irrigation for 12 d (Fig. 3A, B, D34). At this time, all the plants were severely affected by the water deficit as observed by their pale color, dried leaves and wilting phenotype (Fig. 3B, D34). The plants were then returned to regular watering conditions for recovery (Fig. 3A). Fifteen days later, only *OsCPK10HA* plants survived the drought treatment, showing green leaves (Fig. 3B, D49). These results were consistently reproduced in three independent experiments, in which the *OsCPK10HA* plants showed a survival score of 25% to 44%, compared with the complete death of the entire control plant set (Fig. 3C). In two of these experiments, transgenic and wild-type plants were grown in the same pot to ensure that all plants were exposed to the same water deficit (Supplementary Fig. S3A). The decrease in water availability was quantified in all experiments by measuring the soil water moisture (Supplementary Fig. S3B). The improved performance of *OsCPK10HA* plants was also shown as an increment of their fresh weight after recovery in comparison with control plants for a similar dry weight of all the lines (Supplementary Fig. S3C). These results indicate that *OsCPK10HA* expression increases drought tolerance in rice plants.

The observation that all the analyzed plants showed the same wilting phenotype during the drought episode (Fig. 3B, D34) suggests that *OsCPK10HA* plants do have not improved water retention in comparison with control plants. Consistently, the water loss rates of the *OsCPK10HA* plants were not significantly different to those of the control plants (Fig 3D). These results indicate that the improved drought tolerance exhibited by OsCPK10HA plants is not associated to a water loss avoidance mechanism. Instead, OsCPK10 might function to protect cellular structures from the effects of dehydration.

One inevitable consequence of water deficit is the deleterious generation of ROS, which are highly reactive to proteins, lipids and nucleic acids, causing cellular oxidative damage and ultimately cell death (Cruz de Carvalho, 2014; Noctor *et al.*, 2014). To investigate ROS production in *OsCPK10HA* plants, H_2_O_2_ levels were determined in their leaves upon desiccation stress. H_2_O_2_ is the most stable of the major ROS produced during drought (Noctor *et al.*, 2014). *OsCPK10HA* plants accumulated significantly lower levels of H_2_O_2_ during the air-drying treatment than wild-type and EV plants (Fig. 3E).

Various studies reported that ROS-scavenging enzymes are involved in drought tolerance (Badawi *et al.*, 2004; Zhang *et al.*, 2013; Joo *et al.*, 2014). Catalase proteins are among the main H_2_O_2_ scavengers in plant cells (Jiang and Zhang, 2002; Du *et al.*, 2008; Ye *et al.*, 2011). Thus, catalase levels were monitored in *OsCPK10HA* transgenic lines under control conditions or in response to desiccation using western blot analysis. Immunodetection using anti-catalase antibodies showed an immunoreactive polypeptide around 55 kDa, with a higher intensity in the air-dried *OsCPK10HA* leaves than in wild-type or empty vector leaves (Fig. 3F). These results show that the accumulation of OsCPK10HA in rice plants leads to increased catalase accumulation in response to drought stress. As a result, *OsCPK10HA* leaves showed a greater capacity for H_2_O_2_ detoxification and lower H_2_0_2_ levels than control plants (Fig. 3E).

The oxidative damage associated with drought stress causes lipid peroxidation and perturbation of cell membrane function. To further investigate the mechanism underlying drought tolerance in *OsCPK10HA* plants, lipid peroxidation levels were examined in these transgenic plants. Lipid peroxidation was measured as MDA content, MDA being a typical breakdown product of peroxidized polyunsaturated fatty acids in plant membranes (Ayala *et al.*, 2014; Bhattacharjee, 2014). As shown in Fig. 3G, MDA content increased in response to desiccation in all the rice plants but this increment was significantly lower in *OsCPK10HA* plants than in wild-type and empty vector plants. These results show a lower degree of lipid peroxidation caused by desiccation in the *OsCPK10HA* plants, possibly due to their higher ROS detoxifying capacity. We also evaluated the level of membrane damage caused by desiccation by measuring the electrolyte leakage of leaves. Significantly lower values were measured in the *OsCPK10HA* leaves than in wild-type and empty vector plants (Fig. 3H). These results indicate that OsCPK10HA preserves membrane integrity during desiccation stress. Altogether, these observations suggest that OsCPK10HA confers drought tolerance to rice plants by protecting them against the oxidative damage caused by the water deficit.

### OsCPK10HA expression improves oxidative stress tolerance in rice plants

**Fig. 4.**
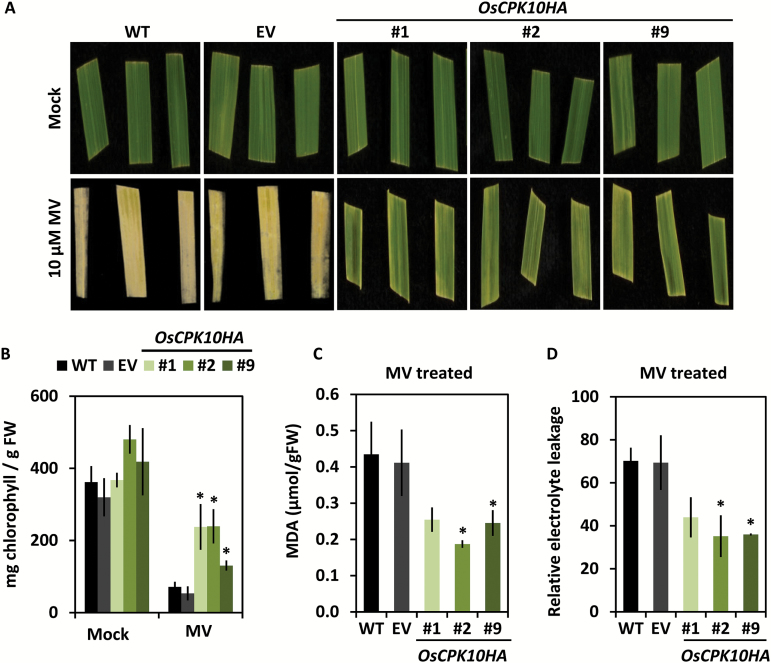
Oxidative stress tolerance of *OsCPK10HA* rice plants. (A) Representative images of leaf fragments of wild-type (WT), empty vector (EV) and *OsCPK10HA* (lines #1, #2, #9) plants treated with mock or 10 µM MV for 4 d. (B) Chlorophyll content of mock leaves or leaves treated with MV for 4 d. Values are the means ± standard error of the mean of three independent assays with four biological replicates each. (C) MDA content, and (D) relative electrolyte leakage of leaf fragments treated with MV for 2 d. Values are the means ± standard deviations of three biological replicates from a pool of three seedlings. Asterisks show significant differences, one-way ANOVA analysis and Tukey test, *P≤0.05. (This figure is available in colour at *JXB* online)

To further investigate the antioxidant capacity of OsCPK10HA plants, leaf pieces of the transgenic and control plants were exposed to the oxidative agent MV. After 4 d of treatment, *OsCPK10HA* leaves remained green whereas control leaves were whitish all over (Fig. 4A). Visual inspection was confirmed by measuring the chlorophyll content of the leaves; *OsCPK10HA* leaves had significantly higher levels than the empty vector or wild-type leaves at the end of the MV treatment (Fig. 4B). These data indicated that the photosynthetic apparatus was less damaged by MV-induced oxidative stress in the lines with accumulated OsCPK10HA. Moreover, a significantly lower amount of MDA was measured in MV-treated *OsCPK10HA* leaves as compared with control leaves (Fig. 4C), denoting lower levels of lipid peroxidation caused by oxidative stress. Consequently, less membrane damage was observed in *OsCPK10HA* leaves than in control leaves, as determined by electrolyte leakage (Fig. 4D). *OsCPK10HA* plants therefore exhibited improved tolerance to oxidative stress mediated by the accumulation of OsCPK10HA.

### OsCPK10HA expression enhances blast disease resistance in rice plants

**Fig. 5.**
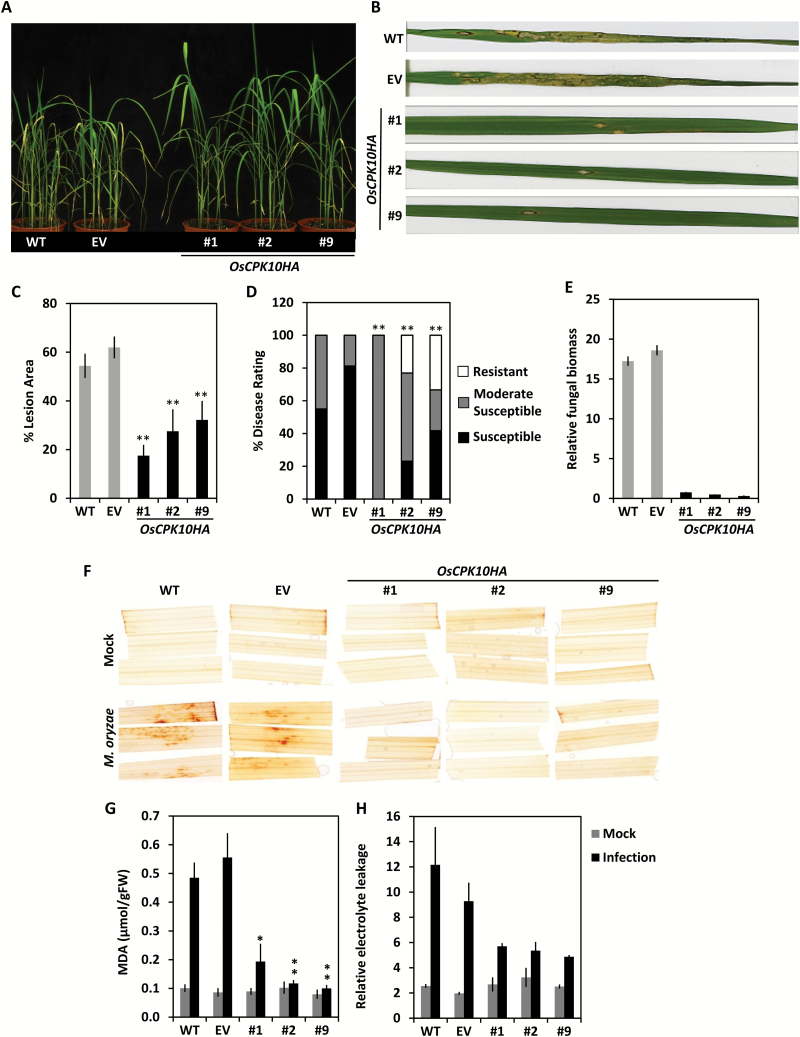
Resistance of *OsCPK10HA* plants to *M. oryzae* infection. (A) Phenotype of wild-type (WT), empty vector (EV) and three independent *OsCPK10HA* rice lines (#1, #2, #9) at 7 d post infection (dpi) with a *M. oryzae* spore suspension of 10^5^ spore/ml. (B) Blast disease leaf symptoms in detail. (C) Average percentage of leaf lesion area. (D) Average percentage of disease rating according to the Standard Evaluation System for Blast Disease (IRRI, 1996). Values are means ± standard error of the mean of two independent assays with 10 plants per line at 7 dpi. Asterisks indicate significant differences, one-way ANOVA analysis, **P<0.01. (E) Relative fungal amount as determined by qPCR of *M. oryzae* 26S rDNA gene compared to rice *OsUbi1* gene. Values are means ± standard deviations of three replicates with 10 plants per line in a pool at 7 dpi. (F) H_2_O_2_ accumulation by DAB staining of rice leaves at 2 dpi with mock or *M. oryzae* spore suspension. (G) MDA content, or (H) relative electrolyte leakage of 2 dpi rice leaves. Values are the means ± standard deviations of three biological replicates from a pool of three seedlings. Asterisks show significant differences, one-way ANOVA analysis and Tukey test, *P≤0.05, **P≤0.01. (This figure is available in colour at *JXB* online)

The effects of *OsCPK10HA* expression in the rice defense response were assessed by inoculating the transgenic plants with the blast fungal pathogen. As shown in Fig. 5A, *OsCPK10HA* plants presented with a healthier appearance at 7 d post-infection (dpi) in comparison to wild-type or empty vector control plants that presented with a wilting phenotype. Close inspection showed extensive necrotic lesions with fungal sporulation on wild-type and empty vector leaves, whereas only a few restricted lesions were present on *OsCPK10HA* leaves (Fig. 5B). The percentage of leaf area affected by blast lesions was determined by image analysis, revealing a statistically significant reduction in lesion area in the three independent transgenic lines as compared with the control leaves (Fig. 5C). Further measures of disease severity showed that a higher percentage of *OsCPK10HA* plants exhibited a resistant phenotype, at around 20%, when compared with wild-type or empty vector plants, none of which were resistant. Also a lower percentage of *OsCPK10HA* plants exhibited a highly susceptible phenotype, at around 20%, when compared with control plants, of which 70% were highly susceptible (Fig. 5D). Consistent with visual inspection, *OsCPK10HA* leaves bore significantly lower fungal biomass than control leaves, as determined by qPCR of *M. oryzae* DNA (Fig. 5E). Collectively, these results show that *OsCPK10HA* positively mediates enhanced resistance to blast disease.

The infection process in the *OsCPK10HA* leaves was then examined by fluorescence microscopy analysis using a *GFP*-expressing *M. oryzae* virulent strain (GFP-Guy11) previously reported (Sesma and Osbourn, 2004; Bundó and Coca, 2016). The fungal infection cycle started similarly on control and *OsCPK10HA* leaves, with spore germination occurring at 6 hpi (Supplementary Fig. S4A, B). Germ tubes and appresoria formation occurred simultaneously on all the inoculated leaves at early infection stages. These appresoria showed similar appearance on control and *OsCPK10HA* leaves (Supplementary Fig. S4C–F). Differences among leaves were observed later at approximately 2 dpi during the more necrothopic phase of the fungal lifecycle (Talbot *et al.*, 2009), when infection lesions become apparent in wild-type and empty vector control leaves (Supplementary Fig. S4G, H) but not in *OsCPK10HA* leaves (Supplementary Fig. S4I, J) . At later stages around 7 dpi, *M. oryzae* completed its lifecycle in control leaves showing the typical blast lesions with bright fluorescent mycelia growing and sporulating (Supplementary Fig. S4K, L), whereas only small necrotic spots were observed in the *OsCPK10HA* leaves (Supplementary Fig. S4M, N). These observations suggest that *OsCPK10HA*-mediated resistance in rice leaves relies on the interference with fungal colonization.

To further investigate the defense mechanism mediated by OsCPK10, we examined the accumulation of H_2_O_2_ in inoculated leaves by 3,3′-diaminobenzidine (DAB) staining. Dark brown patches were observed on wild-type and empty vector leaves throughout the *M. oryzae* inoculated site at 2 dpi, whereas *OsCPK10HA* inoculated leaves did not show any DAB staining and were similar in appearance to the control leaves inoculated with a mock solution (Fig. 5F). This result indicated that the formation of H_2_O_2_ that precedes fungal progression is abolished in *OsCPK10HA* leaves, probably due to their improved antioxidant capacity. Consistently, the level of lipid peroxidation, measured as MDA content was significantly lower in *OsCPK10HA* plants than in control plants upon *M. oryzae* infection (Fig. 5G). Cellular damage, measured as electrolyte leakage, was lower in *OsCPK10HA* leaves than in wild-type and empty vector leaves (Fig. 5H). Altogether, *OsCPK10HA* expression improves resistance to *M. oryzae* infection in rice plants by interfering with fungal progression through enhanced antioxidant activity.

### OsCPK10 is a plasma membrane protein that interacts *in vivo* with catalase A

**Fig. 6.**
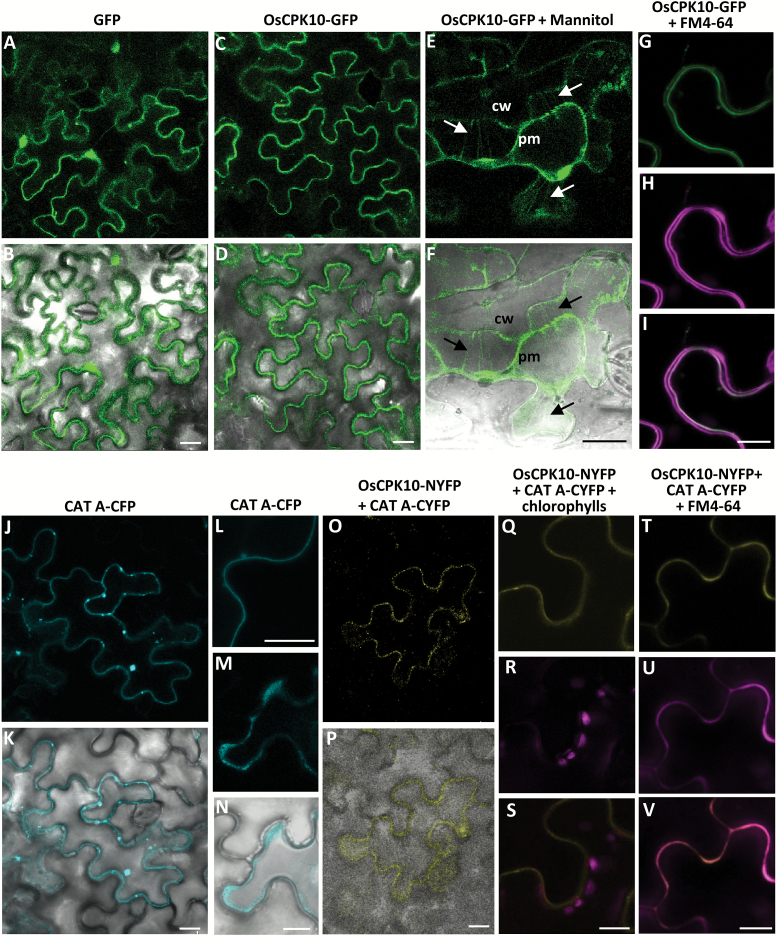
OsCPK10 is localized at the cell plasma membrane and interacts with CAT A. Confocal fluorescence microscopy of *N. benthamiana* epidermal cells transformed with *GFP* (A, B), *OsCPK10-GFP* (C–I), *CAT-CFP* (J–N), or *OsCPK10-NYFP* and *CATA-CYFP* (O–V) via *Agrobacterium*. Images were taken 48 h after agroinfiltration. (E, F) Plasmolysed cell after 15 min of treatment with mannitol. Arrows indicate the Hetchian strands attaching the plasma membrane (pm) to the cell wall (cw). (H, U) Transformed cell stained with the lipophilic dye FM4-64. (A, C, E, G–J, L, M, O, Q–V) Fluorescence images. (B, D, F, K, N, P) Fluorescence and bright field merged images. (I) Merged image of green (G) and magenta (H) fluorescence. (R) Chlorophyll autofluorescence (magenta signals). (S) Merged image of the yellow fluorescence corresponding to the reconstituted YFP and the chlorophyll autofluorescence (magenta). (V) Merged imaged of yellow (T) and magenta (U) fluorescence. Scale bars, 10 µm.

For a better understanding of OsCPK10 function, its subcellular localization was investigated. OsCPK10 possesses the typical four domains of its protein family (Harper *et al.*, 2004; Asano *et al.*, 2005) and has the largest N-terminal variable domain, with 131 amino acid residues, of all rice CPKs. A myristoilation and a palmitoilation site were predicted at position 2 and 5 of its N-terminal domain (NMT-The Myr Predictor, mendel.imp.ac.at/myristate/SUPLpredictor.htm; CSS-Palm 2.0, (Ren *et al.*, 2008). These motifs at the beginning of CPKs have been reported as responsible for their membrane association (Martín and Busconi, 2000; Lu and Hrabak, 2002; Coca and San Segundo, 2010; Witte *et al.*, 2010; Campos-Soriano *et al.*, 2011). To localize OsCPK10 in the plant cell, an *OsCPK10-GFP* fusion gene was transiently expressed in *N. benthamina* leaves via agroinfiltration. Unlike GFP alone, that was ubiquitously distributed inside the epidermal cells (Fig. 6A, B), OsCPK10-GFP was observed at the cell periphery, probably associated with the cell membrane (Fig. 6C–I). The plasma membrane association was demonstrated by treating the leaves with a hypertonic solution of mannitol to induce plasmolysis. OsCPK10-GFP localization remained with the plasma membrane in the shrunken protoplasm, clearly visualized in the typical Hetchian strands that anchor the membrane to the cell wall (Fig. 6E, F). *OsCPK10-GFP* transformed cells were stained with the lipophilic probe FM4-64 that fluoresces intensely upon binding to the plasma membrane. The magenta fluorescence of the FM4-64 staining perfectly overlapped with OsCPK10-GFP green fluorescence (Fig. 6G–I), resulting in the plasma membrane appearing white when the images were merged. These results confirm plasma membrane localization of OsCPK10.

Our results showed that OsCPK10 promotes the accumulation of catalase proteins during drought stress, suggesting that both proteins might function in the same signaling pathway. We therefore speculated of a potential interaction between the two proteins. Three different catalase isoforms have been identified in rice plants, namely CAT A, B and C. CAT B and C have been reported as peroxisomal proteins, whereas CAT A is a cytoplasmic protein (Zhang *et al.*, 2016). Knowing that OsCPK10 is associated with the plasma membrane, catalase A was the most likely catalase to interact with OsCPK10. The proximity between both proteins was confirmed by visualizing CATA-CFP in *N. benthamiana* cells (Fig. 6J–N). The interaction between OsCPK10 and CAT A was addressed using the BiFC assay. Constructs encoding the fusion proteins of OsCPK10 to NYFP and CAT A to CYFP were prepared and used for transient expression in *N. benthamiana* leaves. Fluorescence due to the reconstitution of YFP was observed when both constructs were co-infiltrated (Fig. 6O–V), whereas no fluorescence was detected when single constructs were co-infiltrated or paired with empty vector (pXNGB or pXCGW). The reconstituted fluorescence was visualized at the plasma membrane as shown in the merged images with chlorophyll autofluorescence (Fig. 6Q–S) and with the plasma membrane FM4-64 fluorescence stain (Fig. 6T–V). These results indicate that OsCPK10 physically interacts with CAT A *in planta*.

## Discussion

**Fig. 7.**
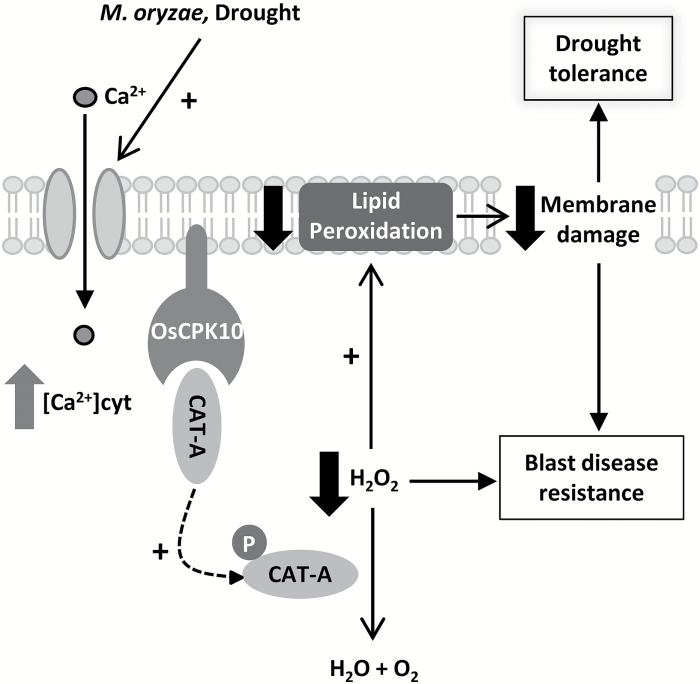
Model of OsCPK10 function in blast disease resistance and drought tolerance. Biotic and abiotic stresses, such as pathogens or drought stress, induce calcium signals that activate plasma membrane-associated OsCPK10. Catalase A protein interacts with OsCPK10, which could activate its enzymatic function by phosphorylation, resulting in H_2_O_2_ detoxification, a reduction in lipid peroxidation and its consequent membrane damage. This protection from oxidative damage mediates blast disease resistance and drought tolerance.

This study reports that the OsCPK10 isoform of the CPK family mediates drought tolerance, as well as blast disease resistance, in rice plants. Here, we show that the constitutive accumulation of this protein in rice plants reduces the accumulation of H_2_O_2_ during desiccation and during the necrotrophic growth phase of *M. oryzae* infection, limiting the oxidative damage associated with both stresses and protecting cellular structures. Together, this leads to improved acclimation to both stressful conditions. A model of OsCPK10 function in context of these stress tolerances is proposed (Fig. 7).

Our results demonstrate that OsCPK10 localizes at the plasma membrane, where proteins perceiving stress signals and calcium channels are also located. Abiotic and biotic stresses are known to trigger calcium signatures (Dodd *et al.*, 2010; Kudla *et al.*, 2010). In our model, we propose that OsCPK10 is activated in response to calcium changes, according to its described calcium dependent kinase activity (Fu *et al.*, 2013). Activation of OsCPK10 then regulates downstream processes. Transgenic *OsCPK10HA* rice plants constitutively accumulate HA-tagged OsCPK10 full-length protein, which contains its calcium-binding regulatory domain and preserves its calcium regulation. Presumably, these plants accumulate OsCPK10HA in an inactive state but ready to be quickly activated by calcium signals upon stress perception. Thus, these plants can mount a fast and strong stress response in all plant tissues when compared with wild-type plants, which require previous transcriptional activation of *OsCPK10* for the accumulation of the protein. In agreement with this suggestion, *OsCPK10* gene induction was detected in wild-type plants in response to drought and *M. oryzae* infection. The transcriptional regulation of *CPK* genes in response to stress has been extensively documented in the literature, which in most cases correlates with its functional involvement in the stress induced response (Coca and San Segundo, 2010; Asano *et al.*, 2012*b*; Fu *et al.*, 2013, 2014; Campo *et al.*, 2014; Bundó and Coca, 2016).

The constitutive accumulation of the OsCPK10 full-length protein therefore enhances stress response in rice plants, whereas under normal growth conditions it seems not to affect plant performance. *OsCPK10HA* plants showed a normal phenotypic appearance, having similar growth, development and yield as wild-type plants. These results suggest that *OsCPK10* overexpression might represent a good strategy for crop improvement.

In our model, OsCPK10 transduces calcium signals into the phosphorylation of downstream components (Fig. 7). The CAT A protein is proposed as an OsCPK10 target based on our results showing their physical interaction at the cell plasma membrane. We also show that OsCPK10 promotes an increased accumulation of catalase proteins in response to desiccation, which is accompanied by a reduction in H_2_O_2_ levels, suggesting that OsCPK10 might regulate catalase activity and stability during water deficit stress. This regulation might be mediated by phosphorylation as reported for Arabidopsis CAT3, whose activity is regulated by AtCPK8 (Zou *et al.*, 2015). A reduction in the accumulation of H_2_O_2_ in *OsCPK10HA* leaves during *M. oryzae* infection was also observed, indicating that the probable OsCPK10-mediated regulation of catalase activity is not only associated with water deficit stress. Moreover, *OsCPK10HA* leaves exhibited enhanced tolerance to the oxidative agent MV. Altogether, OsCPK10HA accumulation confers rice plants with an improved capacity to detoxify reactive H_2_O_2_. This improved antioxidant capacity leads to reduced levels of lipid peroxidation and a better preservation of membrane integrity upon desiccation, oxidative stress and fungal pathogen infection. The oxidation of membrane polyunsaturated fatty acids by excess of ROS associated with different abiotic stresses is known to provoke cell membrane damage and to increase membrane leakage (Wong-ekkabut *et al.*, 2007; Ayala *et al.*, 2014; Bhattacharjee, 2014). Preservation of the integrity and stability of cell membranes is a major determinant of drought tolerance in plants (Bajji *et al.*, 2002; Farooq *et al.*, 2009). In fact, quantitative trait loci of membrane stability have been found in drought tolerant rice (Tripathy *et al.*, 2000). Thus, OsCPK10 seems to assist dehydration tolerance by protecting cellular membranes through an enhanced capacity to detoxify ROS, a widely documented drought tolerance mechanism (Kumar *et al.*, 2014; Nakabayashi *et al.*, 2014; Yin *et al.*, 2015; Fang *et al.*, 2015).

*OsCPK10HA* rice plants also exhibited enhanced resistance to blast disease, as determined by visual inspection, fungal growth quantification and disease lesion measurement. This is in agreement with a previous report that showed that rice plants with constitutively active OsCPK10, formed by deleting its regulatory domains, were more resistant to *M. oryzae* infection (Fu *et al.*, 2013). The enhanced resistance displayed by those plants was associated with the constitutive expression of defense genes (Fu *et al.*, 2013). However transgenic plants constituitively accumulating full-length OsCPK10 did not show consistent and significant changes in the expression of defense marker genes when compared with control plants (Supplementary Fig. S5). These results suggest that *OsCPK10HA* plants do not constitutively activate defenses and that their enhanced protection against blast disease is associated with a different mechanism. Our results suggest that this mechanism is linked to their increased capacity for detoxifying H_2_O_2_ (Fig. 7). Thus, H_2_O_2_ accumulation that precedes fungal progression in wild-type leaves is abolished in *OsCPK10HA* leaves. This H_2_O_2_ accumulation led to increased lipid peroxidation levels, supporting cell death in wild-type plants, in agreement with previous reports (Ohta *et al.*, 1991; Spiteller, 2003). Although cell death might impede the propagation of biotrophic pathogens, it benefits necrotrophic pathogens (Glazebrook, 2005). Considering that *M. oryzae* is a hemibiotrophic fungus, a reduction in lipid peridoxidation, as occurred in *OsCPK10HA* plants, interferes with its fungal necrotrophic growth and therefore leads to resistance to this fungal pathogen.

Collectively, our results demonstrate that the constitutive accumulation of OsCPK10 confers both enhanced drought tolerance and blast disease resistance in rice plants without compromising productivity. Moreover, we show that OsCPK10 function involves enhanced ROS-scavenging activity, which might benefit adaptation to other stresses, since uncontrolled ROS generation is common to most plant stresses. Connections between CPKs and ROS homeostasis have long been established, mainly associated with the positive regulation of ROS production as a signaling molecule in plant defense responses (Romeis *et al.*, 2001; Kobayashi *et al.*, 2007; Boudsocq *et al.*, 2010; Dubiella *et al.*, 2013; Bundó and Coca, 2016). Moreover, rice OsCPK12 was reported to be a negative regulator of ROS production, having an antagonistic role in defense response and abiotic stress signaling (Asano *et al.*, 2012*b*). We demonstrate here that OsCPK10 functions to regulate ROS detoxification. OsCPK10 therefore has a protective role against oxidative damage, which benefits the adaptation of rice plants not only to abiotic stress but also to blast disease. Our studies show that OsCPK10 could be a good molecular target to provide tolerance to multiple distinct stresses in the economically relevant rice crop.

## Supplementary Data

Supplementary data are available at *JXB* online.

Figure S1. OsCPK10HA accumulation in T_0_ transgenic plants.

Figure S2. Performance of *OsCPK10HA* rice plants.

Figure S3. Experimental details on drought tolerance assays with *OsCPK10HA* rice plants.

Figure S4. Microscopic analysis of *Magnaporthe oryzae* infection process on *OsCPK10HA* rice leaves.

Figure S5. Defense marker gene expression in *OsCPK10HA* plants under control conditions.

Table S1. List of primers used in this study

Table S2. Stress-responsive *cis* elements in the 1375 bp upstream region of *OsCPK10* coding sequence.

## Supplementary Material

Supplementary_tables_S1_S2

supplementary_figures_S1_S5

## Abbreviations:

ABA: abscisic acid
CPKs: calcium-dependent protein kinases
dpi: days post-infection
EV: empty vector
H_2_O_2_: hydrogen peroxide
hpi: hours post infection
MDA: malondialdehyde
MV: methylviologen
ROS: reactive oxygen species.
