# Supplementary material for: Calcium-dependent protein kinase OsCPK10 mediates both drought tolerance and blast disease resistance in rice plants
Source: J Exp Bot. 2017 May 2;68(11):2963–75. doi: 10.1093/jxb/erx145 (PMC5853374; doi:10.1093/jxb/erx145)
Supplement: Supplementary_tables_S1_S2 [file erx145_suppl_supplementary_tables_s1_s2.pdf]

Table S1. Primers used in this study. Restriction sites sequences are underlined and HA sequence is in bold.

**Gene name**	**Gene Locus**	**Primer sequence**
OsCPK10HA<br>(qRT-PCR transgene)	LOC_Os03g57450	For 5'ACCCATACGATGTTCCAGATTACG 3'<br>Rev 5'AAATGTTTGAACGATCCCCG 3'
OsCPK10<br>(qRT-PCR)	LOC_Os03g57450	For 5'CAGAACAGTTTCAGCATCGGC 3'<br>Rev 5'CATTTTTTTCCCCGTTTCGAA 3'
OsCPK4<br>(qRT-PCR)	LOC_Os02g03410	For 5' CGTGTGCAGCATGCAGATAA 3'<br>Rev 5' TGATTGCACGTATTCATCGCA 3'
OsCPK20<br>(qRT-PCR)	LOC_Os07g38120	For 5'CCCGTCTTCTCCTACCTTCC 3'<br>Rev 5'CCAGTGGTAAAGCCAACGAC 3'
OsCPK27<br>(qRT-PCR)	LOC_Os12g30150	For 5'GGGATGCCTTGAAGTCACAT 3'<br>Rev 5'TGCACAACAACATTTCCCATA 3'
OsCPK10-CDS + *HA*<br>(cloning to pCAMBIA 1300)	LOC_Os03g57450	For 5'GGATCCAATGGGGAACACGTGCGTC 3'(*BamH*I)<br>Rev 5'GCCCCGGGCTA**AGCGTAATCTGGAACATC**<br>**GTATGGG**TATGGAAGACAACATATCGATCT 3'(*Sma*I)
OsUbi5<br>(qRT-PCR)	LOC_Os01g22490	For 5'TAAGTGCGGCCTCACCTACG 3'<br>Rev 5'GGAGCCTACGCCTAAGCCTG 3'
26S-M.oryzae<br>(qPCR)	AB026819	For 5'TACGAGAGGAACCGCTCATTCAGATAATTA 3'<br>Rev 5'TCAGCAGATCGTAACGATAAAGCTACTC 3'
OSCPK10-CDS<br>(cloning to pMDC85 and pXNGW)	LOC_Os03g57450	For 5'GGATCCGGGAACACGTGCGT 3'(*EcoR*I)<br>Rev 5'GCGGCCGCTGGAAGACAACATATCGAT 3'(*Not*I)
OsCAT-A<br>(cloning to pEarlyGate102 and pXCGW)	LOC_Os02g02400	For 5'CCGAATTCATGGATCCTTGCAAGTTCC 3'(*EcoRI*)<br>Rev 5'CCGATATCTGCTTGGCTTCACGTTGAG 3'(*EcoRV*)
OsPR1b<br>(qRT-PCR)	LOC_Os01g28450	For 5' ACTGCAGCCTGATCCACTCC 3'<br>Rev 5' AGAGGTTCTCGCCAAGGTTGT 3'
OsPR5<br>(qRT-PCR)	LOC_Os12g43380	For 5' GACGACCAGACGAGCACCTT 3'<br>Rev 5' GTCCCTCATGGGCAGAAGAC 3'
OsAOS2<br>(qRT-PCR)	LOC_Os03g12500	For 5' AGCTAGCTAGAAGAGAGTTAGC 3'<br>Rev 5' ACTCGAAGTACTTGTCCTGC 3'
OsJiPR10<br>(qRT-PCR)	LOC_Os03g18850	For 5' AGTTCCTGGACGTGGACAAG 3'<br>Rev 5' CTCGTCCTTCACCTCCACTC 3'
OsEREBP<br>(qRT-PCR)	LOC_Os09g26420	For 5' AAGATCCGTGGCAAGAAAGC 3'<br>Rev 5' GCTTCTGAGCAACAGCTGGTT 3'
OsAccO<br>(qRT-PCR)	LOC_Os09g27750	For 5' CCGGAGTACGTGTTCGGC 3'<br>Rev 5' CCTTCGCATCGAACTTCTGC 3'

Table S2: Stress-responsive c*is* elements in the 1375 bp upstream region of *OsCPK10* coding sequence. The PLACE database (Higo K. et al., 1999, Prestridge D.S., 1991) was used to perform the analysis. The abiotic and biotic stress-related elements are listed by alphabetical order.

Element name	Number of elements	Sequence	Description
ABRELATERD1	2	ACGTG	ABRE-like sequence required for etiolation-induced expression of *erd1* (early responsive to dehydration) in Arabidopsis.
ABRERATCAL	3	MACGYGB	ABRE-related sequence or "Repeated sequence motifs" identified in the upstream regions of 162 Ca(2+)-responsive upregulated genes.<br>M=C/A; Y=T/C; B=T/C/G;
ACGTABREMOTIFA2OSEM	2	ACGTGKC	Experimentally determined sequence requirement of ACGT-core of motif A in ABRE of the rice gene. K=G/T
ACGTATERD1	8	ACGT	ACGT sequence required for etiolation-induced expression of *erd1* in Arabidopsis.
BOXLCOREDCPAL	2	ACCWWCC	Consensus of the putative "core" sequences of box-L-like sequences in carrot (D.c.) PAL1 promoter region. W=A/T
CBFHV	1	RYCGAC	Binding site of barley (H.v.) CBF1 and CBF2; CBFs are also known as dehydration-responsive element (DRE) binding proteins (DREBs). R=A/G; Y=C/T
DPBFCOREDCDC3	2	ACACNNG	A novel class of bZIP transcription factors, DPBF-1 and 2 (Dc3promoter-binding factor-1 and 2) binding core sequence; Dc3 expression is normally embryo-specific, and also can be induced by ABA.
DRE2COREZMRAB17	1	ACCGAC	DRE2 core found in maize (Z.M.) rab17 gene promoter; rab17 is expressed during late embryogenesis, and is induced by ABA.
DRECRTCOREAT	1	RCCGAC	Core motif of DRE/CRT (dehydration-responsive element/C-repeat) cis-acting element found in many genes in Arabidopsis and in rice. R=G/A
GCCCORE	2	GCCGCC	Core of GCC-box found in many pathogen-responsive genes such as PDF1.2, Thi2.1, and PR4; Has been shown to function as ethylene-responsive element.
LTRECOREATCOR15	1	CCGAC	Core of low temperature responsive element (LTRE) of cor15a gene in Arabidopsis (A.t.); ABA responsiveness.
MYB1AT	3	WAACCA	MYB recognition site found in the promoters of the dehydration-responsive gene rd22 and many other genes in Arabidopsis.<br>W=A/T

MYB2CONSENSUSAT	1	YAACKG	MYB recognition site found in the promoters of the dehydration-responsive gene rd22 and many other genes in Arabidopsis. Y=C/T; K=G/T.
MYBCORE	3	CNGTTR	Binding site for all animal MYB and at least two plant MYB proteins ATMYB1 and ATMYB2. ATMYB2 is involved in regulation of genes that are responsive to water stress in Arabidopsis.
MYCCONSENSUSAT	9	CANNTG	MYC recognition site found in the promoters of the dehydration-responsive gene rd22 and many other genes in Arabidopsis; This sequence is also known as RRE (Response element). N=A/T/G/C
PROXBBNNAPA	1	CAAACACC	"prox B (proximal portion of B-box) found in napA gene of *Brassica napus* (B.n.); Required for seed specific expression and ABA responsiveness; ABRE mediated transactivation by ABI3 adn ABI3-dependent response to ABA
SEBFCONSSTPR10A	2	YTGTCWC	Binding site of the potato silencing element binding factor (SEBF) gene found in promoter of pathogenesis-related gene (PR-10a); Located between -45 and -39; Similar to the auxin response element; W=A/T, Y=C/T
WBBOXPCWRKY1	1	TTTGACY	WRKY proteins bind specifically to the DNA sequence motif (T)(T)TGAC(C/T), which is known as the W box; Found in amylase gene in sweet potato, alpha-Amy2 genes in wheat, barley, and wild oat, PR1 gene in parsley, and a transcription factor gene in Arabidopsis; Y=C/T
WBOXATNPR1	1	TTGAC	"W-box" found in promoter of *Arabidopsis thaliana* (A.t.) *NPR1* gene; They were recognized specifically by salicylic acid (SA)-induced WRKY DNA binding proteins
WBOXNTCHN48	1	CTGACY	W box identified in the region between -125 and -69 of a tobacco class I basic chitinase gene CHN48; NtWRKY1, NtWRKY2 and NtWRKY4 bound to W box; NtWRKYs possibly involved in elicitor-respsonsive transcription of defense genes in tobacco; Y=C/T
WBOXNTERF3	5	TGACY	W box found in the promoter region of a transcriptional repressor ERF3 gene in tobacco; May be involved in activation of ERF3 gene by wounding. Y=C/T.
WRKY71OS	7	TGAC	A core of TGAC-containing W-box; Binding site of rice WRKY71, a transcriptional repressor of the gibberellin signaling pathway; Parsley WRKY proteins bind specifically to TGAC-containing W box elements within the Pathogenesis-Related Class10 (PR-10) genes.
