# Supplementary material for: Calcium-dependent protein kinase OsCPK10 mediates both drought tolerance and blast disease resistance in rice plants
Source: J Exp Bot. 2017 May 2;68(11):2963–75. doi: 10.1093/jxb/erx145 (PMC5853374; doi:10.1093/jxb/erx145)
Supplement: supplementary_figures_S1_S5 [file erx145_suppl_supplementary_figures_s1_s5.pdf]

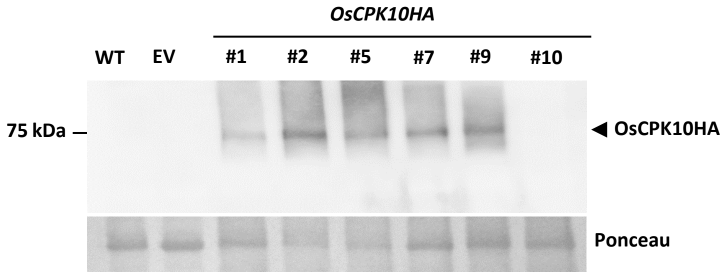


**Figure S1. OsCPK10HA accumulation in $T_0$ transgenic plants.** Immunoblot analysis of protein extracts from leaves of wild-type (WT), empty vector (EV) and the indicated *OsCPK10HA* transgenic lines using specific anti-HA antibodies.

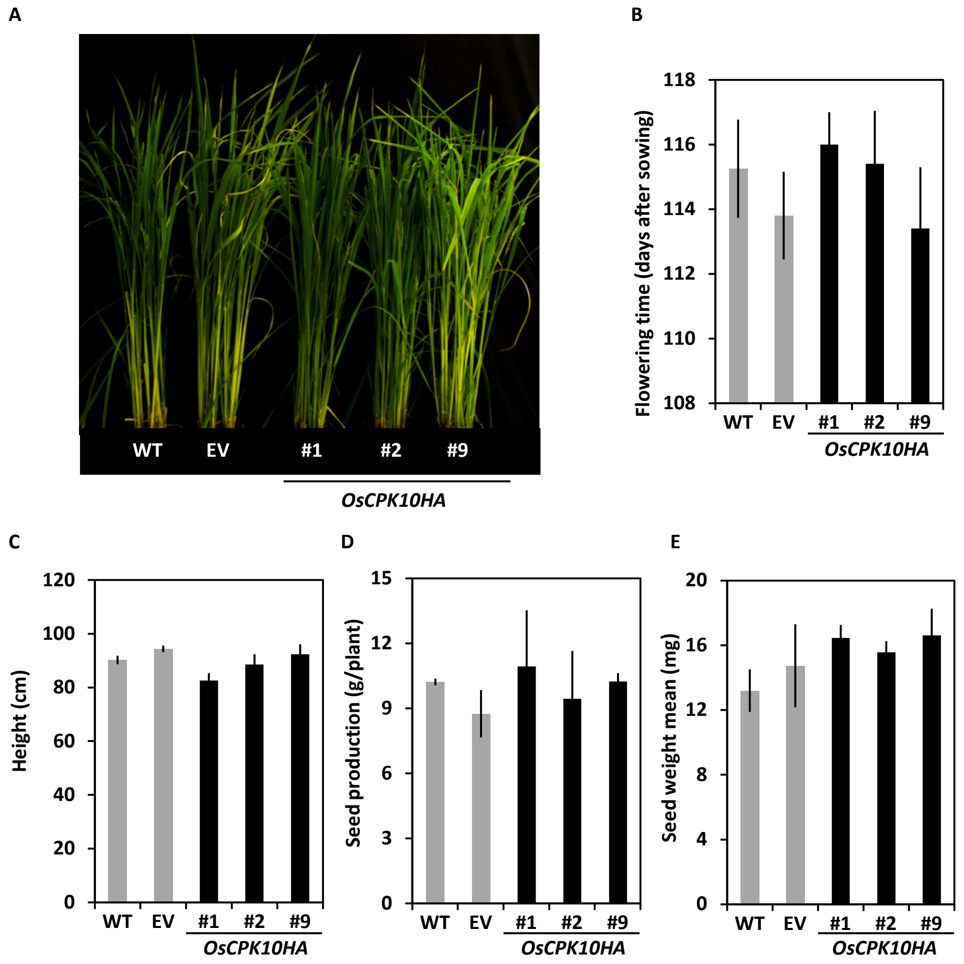


**Figure S2. Performance of *OsCPK10HA* rice plants.** (A) Phenotypic appearance of wild-type (WT), empty vector (EV) and *OsCPK10HA* (lines #1, #2 and #9) rice plants at 127 days after sowing. (B) Flowering time average in days after sowing . (C) Plant height average at heading time. (C) Average of grain yield per plant grown under randomized distribution. (E) Seed weight average. Values are the mean ± SEM of 3 independent assays in which five plants per line were analyzed. No significant differences were observed for any of these parameters.

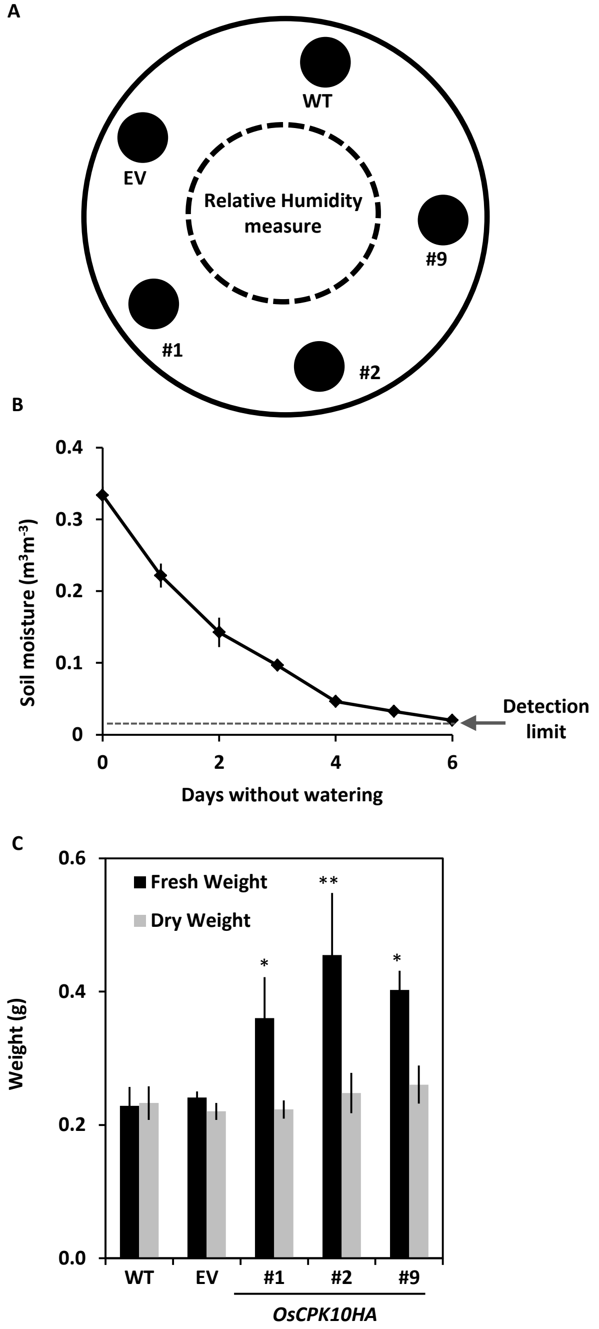


**Figure S3. Experimental details on drought tolerance assays with *OsCPK10HA* rice plants.** (A) Scheme of the plants distribution during the drought tolerance assays. (B) Mean values of the soil moisture of the different pots exposed to drought as measured by HH2 Moisture meter 2.3 (Delta-T devices Ltd.). Arrow indicates the device detection limit. (C) Fresh and dry weights of *OSCPK10HA* and control plants after rewatering (D49). Values are the means ± SEM of three independent assays with five plants per line. Asterisks show significant differences (one-way ANOVA analysis, *P≤0.05, **P≤0.01).

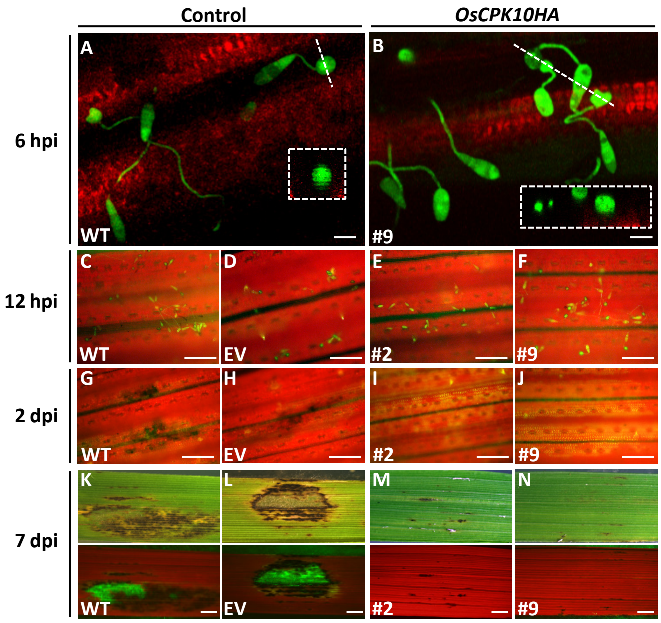


**Figure S4**. **Microscopic analysis of *Magnaporthe oryzae* infection process on *OsCPK10HA* rice leaves.** Representative images of *OsCPK10HA* (lines #2 and #9), empty vector (EV) and wild-type (WT) leaves, inoculated with the GFP-*M. oryzae* spores ($10^5$ spores/ml). (A-B) Confocal laser microscopic images of leaves at 6 hpi, corresponding to projections, and inset boxes to xz slides. Epifluorescence images at 12 hpi (C-F), 2 dpi (G-J) or 7dpi (K-N, lower panels). (K-N, upper panels) Steroscopic brightfield images. Bars = 10 µm (A-B), 50 µm (C-J), 1 mm (K-N).

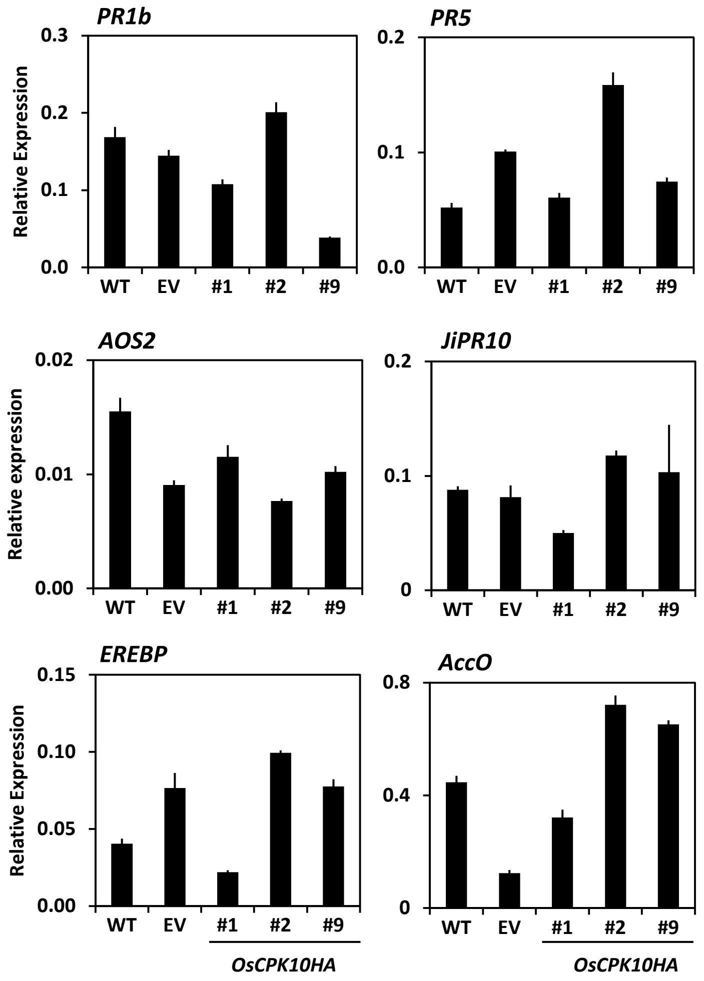


**Figure S5. Defense marker gene expression in *OsCPK10HA* plants under control conditions.** Transcript levels of different salicylic acid, jasmonic acid and ethylene-mediated defense marker genes in leaves of wild-type (WT), empty vector (EV) and three independent *OsCPK10HA* lines (#1, #2 and #9) under control conditions, determined by qRT-PCR analysis and normalized to *OsUbi5* transcript levels. Values are the means and SD of three replicates.
